# Community differentiation of bacterioplankton in the epipelagic layer in the South China Sea

**DOI:** 10.1002/ece3.4064

**Published:** 2018-04-19

**Authors:** Yi Zhang, Jie Li, Xuhua Cheng, Yinfeng Luo, Zhimao Mai, Si Zhang

**Affiliations:** ^1^ CAS Key Laboratory of Tropical Marine Bio‐resources and Ecology South China Sea Institute of Oceanology Chinese Academy of Sciences Guangzhou China; ^2^ State Key Laboratory of Tropical Oceanography South China Sea Institute of Oceanology Chinese Academy of Sciences Guangzhou China; ^3^ Beijing Institute of Genomics Chinese Academy of Sciences Beijing China

**Keywords:** bacterioplankton community, epipelagic layer, functional traits, longitudinal distribution, pyrosequencing, stratification distribution

## Abstract

The South China Sea (SCS) is the largest marginal sea in the western tropical Pacific Ocean and is characterized by complex physicochemical environments. To date, the biogeographic patterns of the microbial communities have rarely been reported at a basin scale in the SCS. In this study, the bacterial assemblages inhabiting the epipelagic zone across 110°E to 119°E along 14°N latitude were uncovered. The vertical stratification of both bacterial taxa and their potential functions were revealed. These results suggest that the water depth‐specific environment is a driver of the vertical bacterioplankton distribution. Moreover, the bacterial communities were different between the eastern stations and the western stations, where the environmental conditions were distinct. However, the mesoscale eddy did not show an obvious effect on the bacterial community due to the large distance between the sampling site and the center of the eddy. In addition to the water depth and longitudinal location of the samples, the heterogeneity of the phosphate and salinity concentrations also significantly contributed to the variance in the epipelagic bacterial community in the SCS. To the best of our knowledge, this study is the first to report that the variability in epipelagic bacterioplankton is driven by the physicochemical environment at the basin scale in the SCS. Our results emphasize that the ecological significance of bacterioplankton can be better understood by considering the relationship between the biogeographic distribution of bacteria and the oceanic dynamics processes.

## 1 INTRODUCTION

Microbial plankton are the central members involved in energy and matter fluxes in the sea and play a fundamental role in biogeochemical cycles (Azam et al., 1983; DeLong et al., 2006; Karl, 2002). These marine microorganisms respond to variations in the physicochemical and biologic forcing in nature (Doney et al., 2012). Depth has been considered a primary factor that influences the composition of the marine microbial community and leads to stratification (DeLong et al., 2006; Field et al., 1997; Gordon & Giovannoni, 1996). In the epipelagic water column, gradients of light quality and intensity (Giovannoni & Stingl, 2005), latitude (Fuhrman et al., 2008), temperature, salinity (Gilbert et al., 2009), and resources such as macronutrient and trace metal concentrations (Giovannoni & Stingl, 2005) have been reported to drive the variations in microbial communities. On the other hand, the hydrography of the ocean also has a strong impact on the distribution of marine bacterioplankton (Baltar, Aristegui, Gasol, Lekunberri, & Herndl, 2010; Chelton, Schlax, & Samelson, 2011; Galand, Potvin, Casamayor, & Lovejoy, 2010; McGillicuddy et al., 1998, 1999; Nelson, Carlson, Ewart, & Halewood, 2014). Water mixing due to winds, waves, and currents has been proposed to impact the dispersal of marine bacterioplankton (Galand et al., 2010). For example, mesoscale eddies (∼200 km diameter), which have been commonly found in oligotrophic gyres, can change the microbial community structure by facilitating spatially and temporally transient pulses of nutrients and shifting density isoclines (McGillicuddy et al., 1998, 1999; Nelson et al., 2014). In addition, monsoons also act as an environmental forcing factor and drive changes in the phytoplankton community (Brown et al., 2002; Campbell et al., 1998). Research on the changes in marine microbial communities in response to spatial and environmental variations is central to determining the factors that structure the community distribution and further enhances our predictive understanding of the dynamic patterns of microbial biogeochemical cycling (Giovannoni & Vergin, 2012; Nelson, 2009).

The South China Sea (SCS) is characterized by a tropical and subtropical climate and is the largest marginal sea in the western tropical Pacific Ocean. It is a semienclosed deep basin with a maximum depth of over 5,000 m (Chen, Wang, Wang, & Pai, 2001). The deep central SCS is permanently oligotrophic and has complex physical environments (Wang, Su, & Chu, 2003; Wong, Ku, Mulholland, Tseng, & Wang, 2007).

Mesoscale eddies are a typical physical process in the SCS (Wang et al., 2003). Phytoplankton productivity has been confirmed to be influenced by the nutrient flux driven by eddies (He, Zhan, Cai, & Li, 2016; He et al., 2017; Huang, Hu, Xu, Cao, & Wang, 2010; Liu, Tang, Huang, & Yin, 2017; Wang, Huang et al., 2016). In addition, the distribution of the most abundant picophytoplankton groups, such as *Prochlorococcus* and *Synechococcus*, was found to be significantly influenced by warm or cold eddies (Jing & Liu, 2012; Wang, Tan et al., 2016). Bacterial communities have also been found to be influenced by cyclonic (Zhang, Jiao, Sun, Hu, & Zheng, 2011; Zhang, Zhao, Sun, & Jiao, 2011) and anticyclonic (Li et al., 2014) eddy perturbations. For example, the distributions of some deep‐water groups were found to exhibit isopycnal uplift (Zhang, Jiao et al., 2011), and diazotrophic *Proteobacteria* were found to attain new nitrogen from mesoscale cyclonic eddies, which contributed to nitrogen fixation (Zhang, Zhao et al., 2011). Due to the limited sampling sites and depths, a clear conclusion on the effects of mesoscale eddies on bacterial communities has not been reached. Aside from the studies mentioned above (Li et al., 2014; Zhang, Jiao et al., 2011; Zhang, Zhao et al., 2011), the effects of eddies on the bacterial community structure have rarely been studied in the SCS.

Multiscale physical processes that are mainly driven by monsoonal winds create the complex circulations in the SCS (Su, 2004). The upper layer circulation in the SCS displays a strong seasonal cycle in response to the southwest monsoon in the summer and the northeast monsoon in the winter (Qu, 2000). The SCS is divided into two parts (northwest and southeast) and forms two distinct environmental conditions separated by the axis of the maximum monsoonal winds, which are northeasterly in the winter (Liu, Jiang, Xie, & Liu, 2004) and southwesterly in the summer (Xie, Xie, Wang, & Liu, 2003). As microorganisms respond to environmental forcing, it is reasonable to hypothesize that the distribution of the bacterial community is associated with the differentiation between the eastern and western environments. Nevertheless, the structures of the microbial taxa and functions have not been investigated at a basin scale in the SCS.

In this study, we sought to uncover the bacterial assemblages in the epipelagic layer across 110°E to 119°E along 14°N latitude. High‐throughput pyrosequencing allows for efficient DNA sequencing of the microbial populations and provides a comprehensive description of both the richness and diversity of the microbial populations present in the environment (Huber et al., 2007; Huse, Huber, Morrison, Sogin, & Mark Welch, 2007; Ronaghi, 2001; Sogin et al., 2006). Thus, we analyzed the bacterial community profiles using data from pyrosequenced amplicons of the hypervariable V1–V3 regions of the 16S ribosomal RNA gene from 43 samples collected across 11 epipelagic water depth profiles (5–200 m) along the 14°N transect. Based on the relationship between phylogenic affiliation and biologic function, PICRUSt (phylogenetic investigation of communities by reconstruction of unobserved states; Langille et al., 2013) was further applied to predict the functional gene composition from the phylogenetic data of the bacterial communities. We investigated the drivers of the distribution of the bacterioplankton populations and the functional traits across the epipelagic layers along the 14°N transect by coupling these community profiles with spatial and physicochemical environmental variables.

## 2 MATERIALS AND METHODS

### 2.1 Study sites and sampling

**Figure 1:**
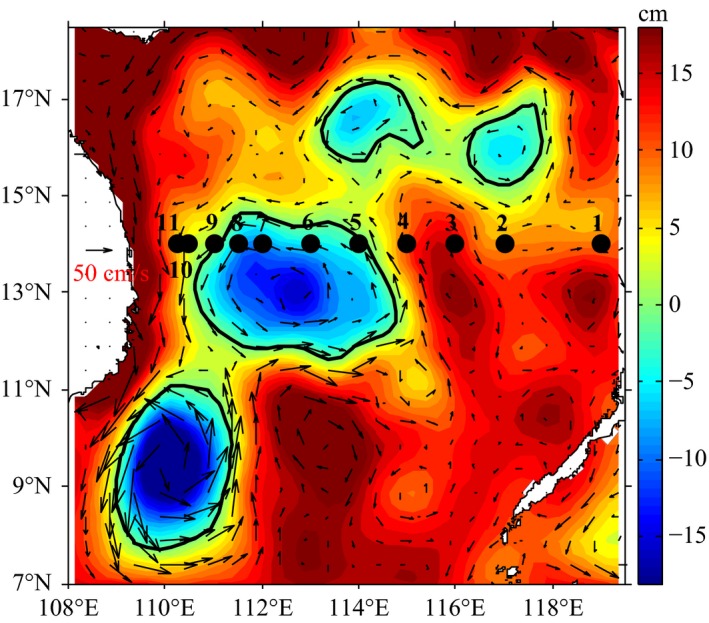
Sea surface map of the 11 sampling stations where seawater samples were collected during the cruise in the northern basin of the South China Sea. The points are profile locations labeled with code numbers

Sampling was conducted along a 14°N transect during a cruise in the SCS from 19 to 22 October 2012. Seawater samples were collected from depths of 5, 25, 75, and 200 m at a total of 11 stations; however, no sample was collected from 200 m at station no. 8 (Table S1, Figure 1). Seawater samples and temperature and salinity records were collected from a CTD‐General Oceanic Rosette sampler with Niskin bottles (SBE 9 plus, SeaBird, USA). After the samples were filtered through 20‐μm bolting cloths to remove the zooplankton and other contamination, one liter of seawater from each collection was filtered through a 0.22‐μm polycarbonate filter (Millipore). The filters were rolled with sterilized forceps and placed in 2‐ml cryogenic vials (Corning), subsequently frozen at −20°C, and stored long term at −80°C until further processing in the laboratory. The seawater samples for the inorganic nutrient assays (nitrate, nitrite, phosphate, and silicate) were collected through silk yarn filters and immediately stored at −20°C until further processing at the Marine Environmental Engineering Center at the South China Sea Institute of Oceanology, Chinese Academy of Sciences. Sea surface height during the sampling cruise was identified in the laboratory from near‐real‐time satellite altimetry observations (AVISO Live Access Server: http://las.aviso.oceanobs.com/).

### 2.2 DNA extraction, PCR amplification, and pyrosequencing

The total DNA was extracted using the PowerWater^®^ DNA Isolation Kit (MoBio, Solana Beach, CA, USA) according to the manufacturer's instructions. Hypervariable V1–V3 regions of the bacterial 16S ribosomal RNA gene were amplified for all samples using the bacterial forward primer 27F (5′–AGAGTTTGATCCTGGCTCAG–3′) (Hongoh, Ohkuma, & Kudo, 2003) with the 5′ Roche FLX Amplicon A adaptor CCTATCCCCTGTGTGCCTTGGCAGTC followed by a TCAG linker on the 5′ end and the reverse primer 534R (5′–ATTACCGCGGCTGCTGG–3′) (Nossa et al., 2010) with the 5′ Roche FLX Amplicon B adaptor CCATCTCATCCCTGCGTGTCTCCGACTCAG followed by a TCAG linker and a unique oligonucleotide 10‐base pairs (bp) barcode (Hamady, Walker, Harris, Gold, & Knight, 2008) on the 5′ end. The 50 μl compounds of the PCR amplifications, which were performed in a Mastercycler Pro (Eppendorf, Hamburg, Germany), were 4 μl of 2.5 mmol/L deoxynucleotide triphosphate mixture (TaKaRa), 1 μl of each of 10 μmol/L coupled primers, 5 μl (25–50 ng) of template DNA, 2.5 μl of *Ex Taq* DNA polymerase [TaKaRa Biotechnology (Dalian) Co., Ltd., China], 5 μl of accessory buffer, and 31.5 μl of sterilized water. The conditions of the touchdown PCR amplifications were 300 s at 94°C; 30 cycles of 60 s at 94°C, 60 s at 60°C (decreasing by 0.5°C in each cycle), and 60 s at 72°C; and a final extension for 600 s at 72°C. Each genomic DNA sample was amplified in triplicate. The amplicons with the same barcode were pooled and purified using a QIAquick^®^ Gel Extraction Kit (QIAGEN, Germany). The quality of the purified PCR products was assessed using a NanoDrop spectrophotometer (Thermo Scientific, Vantaa, Finland). Pyrosequencing was run on the Roche 454 Genome Sequencer FLX System (Roche, Nutley, NJ, USA) at the Beijing Institute of Genomics, Chinese Academy of Sciences.

### 2.3 Sequence processing and analysis

The 16S rRNA gene amplicon sequencing data were analyzed using Mothur (version 1.34.4; Schloss et al., 2009). The amplicon libraries from the samples were demultiplexed to minimize the impact of potential sequencing errors. The criteria used to retain the sequences were the following: sequence length >200 bp, no ambiguous bases, homopolymers less than 8 bp, and application of a quality window of 50 bp with an average flowgram score >25. Chimeras were eliminated from the remaining reads by running the chimera.uchime command. The qualified reads were identified using the classify.seqs command with the SILVA reference files (Seed_v119) and a bootstrap confidence level of 80%. Groups that were considered contaminants, including mitochondria, chloroplast, Archaea, Eukaryota, and unknown, were removed, as described in Schloss, Gevers, and Westcott (2011). The clustering of the sequences into operational taxonomic units (OTUs) was performed using the OptiClust algorithm with a 97% sequence similarity threshold. The taxonomic affiliation of each OTU was inferred using the classify.otu command implemented in Mothur (Schloss et al., 2011).

### 2.4 Statistical analyses

The datasets were standardized by subsampling with the lowest sequence number (9,333 in this study) and randomly selecting from each library 1,000 times. The species richness and diversity estimates were calculated with Mothur (version 1.34.4). The Bray–Curtis distance matrix was estimated based on the relative abundances of the subsampled OTUs. Hierarchical clustering (CLUSTER; Clarke, 1993) was run using PRIMER 5 software (PRIMER E Ltd, UK). Significant differences among the bacterial communities were verified through an analysis of similarities (ANOSIM). The Bray–Curtis distance matrix was used to generate one‐way ANOSIM statistics with 999 permutations (Clarke, 1993).

The correlations between bacterial community composition, detailed bacterial genera, and environmental variables were investigated by a canonical redundancy analysis using the CANOCO 4.5 software package for Windows (Leps, Smilauer, Leps, & Smilauer, 2003). The species data and environmental data, including temperature, salinity, nitrate, nitrite, phosphate, silicate, depth, and location (Table S1), were input and standardized. The location was identified by calculating the longitude‐based relative distance from sampling station no. 1. A detrended correspondence analysis (DCA) with detrending by segments was used to determine the lengths of the community composition gradients. The gradients were below two units, so a redundancy analysis (RDA) was applied to determine the correlation between the explanatory variables and the community composition using Monte Carlo permutation tests (499 unrestricted permutations).

Differences in environmental factors among samples were tested using the Student's *t*‐test. Differences in functional gene compositions among water depths were tested with Kruskal–Wallis test follow by a multiple comparison test using the pgirmess package in R (Siegel & Castellan, 1988; https://cran.r-project.org/web/packages/pgirmess/index.html).

### 2.5 Functional genes predicted with PICRUSt

PICRUSt is genome prediction bioinformatics software based on 16S rRNA gene data ( http://picrust.github.com/picrust/). To use the tool to calculate the functional information of the microbial OTUs, a synthetic OTU table, in which the OTUs were assigned at 97% similarity and mapped to the Greengenes (version 13.5) database, was created for functional prediction using the make.biom command in Mothur (version 1.34.4). The synthetic table was uploaded to the online PICRUSt pipeline on the Galaxy website ( http://huttenhower.sph.harvard.edu/galaxy/root?tool_id=PICRUSt_normalize) and was then normalized using the normalize by copy number module. The metagenome predictions were analyzed to produce a virtual metagenome of the Kyoto Encyclopedia of Genes and Genomes (KEGG) ortholog abundances for each sample in the given OTU table using the predict metagenome module. In addition, the categorize by function module was utilized to examine a specified level for metagenome predictions and count the genes for each pathway.

## 3 RESULTS

### 3.1 Hydrographic physical and chemical characteristics

The temperature, salinity, and inorganic matter constituents, including nitrate, phosphate, and silicate, of the seawater showed vertical patterns with water depth, and the upper epipelagic 5‐m and 25‐m layers were strongly mixed (Table S1, Figures S1 and S2). The satellite observations showed that the sea surface height in the eastern SCS was higher than that in the western SCS (Figure 1). In addition, an anticyclonic eddy and a cyclonic eddy were identified adjacent to the 14°N transect during sampling (Figure 1). Sampling stations 1–4 were located to the north of the anticyclonic eddy, and stations 5–8 were located to the north of the cyclonic eddy. The distances between the centers of the eddies and the 14°N transect were greater than 300 km.

Similar to the observations in sea surface height, the temperatures were higher and the salinities were lower at stations 1–4 in the east than at stations 5–11 in the west throughout the epipelagic waters (*p* < .023, *t*‐test), especially at the depth of 75 m (*p *< .004, *t*‐test; Table S1, Figure S1). In addition, the concentrations of phosphate, nitrate, and silicate at 75 m were significantly higher at stations 5–11 than at stations 1–4 (*p* < .012, *t*‐test; Table S1, Figure S1); conversely, the nitrite concentrations were higher at stations 1–4 than at stations 5–11 (*p *= .006, *t*‐test; Table S1, Figure S1). The phosphate concentrations in the 200‐m layer were also higher at stations 5–11 than at stations 1–4 (*p *= .02, *t*‐test). The cluster analysis of the physical and geochemical parameters further demonstrated the distinct environmental conditions of stations 1–4 and stations 5–11 at 75 m (Figure S2).

### 3.2 16S rRNA gene pyrosequencing and bacterial diversity

**Figure 2:**
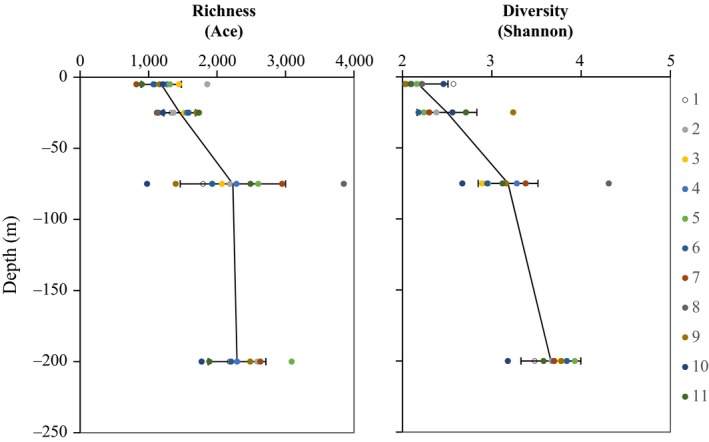
Richness and diversity of the bacterioplankton increase with depth. Each panel shows the depth distribution of each variable (mean ± one standard deviation) across all 11 sampling profiles. The different color circles indicate the samples at different sites

Forty‐three samples were analyzed through 16S rRNA gene amplicon pyrosequencing. The pyrosequencing produced a total of 802,618 valid quality‐filtered reads following denoising and alignment trimming. These sequences were clustered into 7,146 OTUs at a 3% dissimilarity level, and 3,410 of these OTUs were represented by more than one sequence. There were 319–1,319 OTUs identified in each of the 43 libraries, and the number of OTUs increased with increasing water depth (Table 1). The rarefaction curves (with a 3% cutoff value) tended to approach the saturation plateau, indicating that the sampling sizes were sufficient (Figure S3). Further, the coverage estimation of each sample proved that 96.2%–99.1% of the species were obtained (Table 1). The Shannon, abundance coverage estimator (ACE), and Chao1 indices ranged from 1.93 to 4.31, 821.42 to 3851.57, and 599 to 2739.32, respectively (Table 1). Moreover, both bacterial richness and diversity obviously increased in response to increasing water depth throughout the basin and at most of the individual sampling sites. However, the richness at stations 3, 7, and 11 increased up to the depth of 75 m and then decreased to the depth of 200 m, and the richness at stations 9 and 10 decreased up to the depth of 75 m and then increased to the depth of 200 m (Figure 2).

**Table 1:** Sequencing information and diversity estimates (at 97% similarity) for the 43 samples collected from the South China Sea

Index	No. of Seqs	Coverage	OTUs	Chao 1	ACE	Shannon	Simpson
A1	11,675	0.98	424	821.22	1091.05	2.57	0.21
A2	29,502	0.99	595	1267.04	1857.86	2.21	0.26
A3	24,576	0.99	477	1019.71	1443.73	2.1	0.26
A4	20,277	0.99	448	843.28	1267.34	2.1	0.26
A5	21,386	0.99	521	989.13	1312.18	2.16	0.31
A6	22,224	0.99	416	767.67	1071.55	2.03	0.29
A7	17,602	0.99	319	599	821.42	1.93	0.29
A8	15,240	0.99	366	677.68	903.61	2.22	0.26
A9	16,889	0.99	376	767.42	1160.05	2.04	0.28
A10	19,661	0.99	445	821.64	1208.17	2.46	0.21
A11	19,570	0.99	390	675	902.17	2.09	0.28
B1	24,396	0.99	480	1008.68	1332.36	2.29	0.25
B2	18,795	0.99	496	944.99	1364.3	2.38	0.23
B3	22,383	0.99	589	1110.68	1519.05	2.56	0.22
B4	29,327	0.99	612	1111.69	1558.68	2.3	0.25
B5	28,514	0.99	601	1231.65	1739.9	2.24	0.28
B6	15,396	0.98	440	1100.2	1585.41	2.18	0.29
B7	16,261	0.99	443	815	1122.63	2.3	0.29
B8	14,894	0.99	426	765.65	1142.75	2.71	0.18
B9	15,286	0.98	539	1121.41	1697.85	3.24	0.11
B10	18,723	0.99	507	913.72	1210.07	2.56	0.22
B11	20,311	0.98	595	1180.53	1728.62	2.71	0.19
C1	16,142	0.98	640	1362.93	1796.79	2.9	0.2
C2	22,680	0.98	710	1413.39	2187.9	2.96	0.16
C3	16,587	0.98	671	1401.29	2073.25	2.89	0.17
C4	20,182	0.98	795	1545.61	2284.52	3.28	0.15
C5	24,702	0.98	949	1758.49	2603.18	3.38	0.12
C6	15,384	0.98	586	1264.84	1930.14	2.95	0.15
C7	22,882	0.98	935	1957.16	2950.48	3.38	0.12
C8	31,170	0.98	1319	2729.32	3851.57	4.31	0.04
C9	11,673	0.98	464	909.5	1395.79	3.16	0.11
C10	16,077	0.99	357	683.53	979.06	2.67	0.15
C11	20,683	0.98	803	1777.61	2488.49	3.12	0.14
D1	17,544	0.98	852	1581.46	2181.33	3.48	0.12
D2	17,126	0.97	921	1899.01	2588.97	3.67	0.11
D3	9,333	0.96	664	1306.89	1773.89	3.77	0.09
D4	12,817	0.97	771	1646.35	2293.64	3.69	0.1
D5	15,145	0.97	908	2063.39	3090.54	3.93	0.07
D6	11,867	0.96	786	1566.39	2205.73	3.84	0.08
D7	15,405	0.97	902	1777.9	2632.74	3.7	0.1
D9	13,581	0.97	710	1671.45	2485.37	3.78	0.07
D10	17,707	0.98	600	1186.08	1775.82	3.18	0.1
D11	11,043	0.97	646	1240.12	1887.33	3.58	0.11

A represents 5 m depth; B represents 25 m depth; C represents 75 m depth; D represents 200 m depth; numbers 1–11 represent the stations.

### 3.3 Bacterial community structure

Twenty‐three bacterial phyla were identified in the epipelagic bacterial communities, including nineteen formally described phyla and four candidate phyla (Figure S4). Several unclassified reads were found in all libraries with proportions ranging from 0.46% to 8.73%.

**Figure 3:**
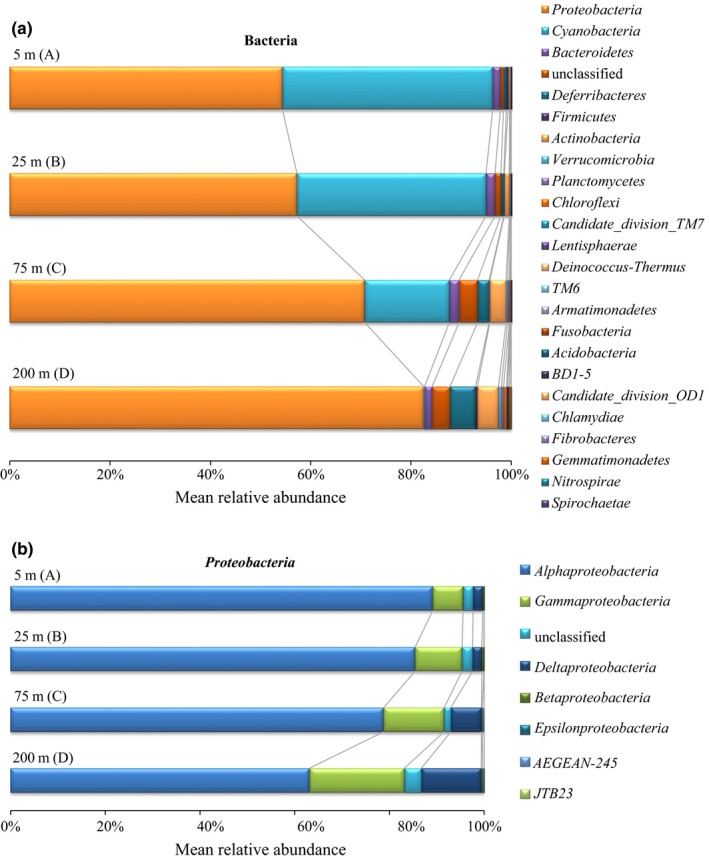
Mean relative abundances of the bacterial groups at each depth (5 m, *n* = 11; 25 m, *n* = 11; 75 m, *n* = 11; 200 m, *n* = 10), (a) on the phylum level, and (b) on the class level within *Proteobacteria*

*Proteobacteria* dominated all libraries (accounting for 41.94%–92.62%), and the proportion of *Proteobacteria* (mean value of the relative abundance at each depth) increased with depth (Figure 3a). Within *Proteobacteria*,* Alphaproteobacteria* were dominant and ubiquitous in all samples and showed decreasing abundances with increasing depth (Figure 3b). Most reads in *Alphaproteobacteria* were affiliated with SAR11 (accounting for 12.41%–91.43% within *Alphaproteobacteria*). In addition to SAR11, *Rhodobacterales*,* Rickettsiales*, and *Rhizobiales* were the other major groups within *Alphaproteobacteria*. In contrast to the distribution of *Alphaproteobacteria*,* Gammaproteobacteria* and *Deltaproteobacteria* were more abundant in the deeper epipelagic layers (Figure 3b). *Oceanospirillales* and SAR86 were the major *Gammaproteobacteria* groups, while *Pseudomonadales* contributed to the largest amount of *Gammaproteobacteria* at stations 9 and 10. The reads affiliated with SAR324 accounted for 45.71%–94.06% of *Deltaproteobacteria* in all samples (Figure 3b).

The relative abundances of *Cyanobacteria* were greater than 39% in the surface seawater, and they dramatically decreased to less than 1% at 200 m (Figure 3a). Both *Prochlorococcus* and *Synechococcus* were the major *Cyanobacteria* at the 5 and 25 m depths. The major groups *Actinobacteria* and *Deferribacteres* were enriched in the subsurface water layers, that is, 75 and 200 m, while *Bacteroidetes* was relatively evenly distributed across multiple depth layers in terms of the phylum level.

**Figure 4:**
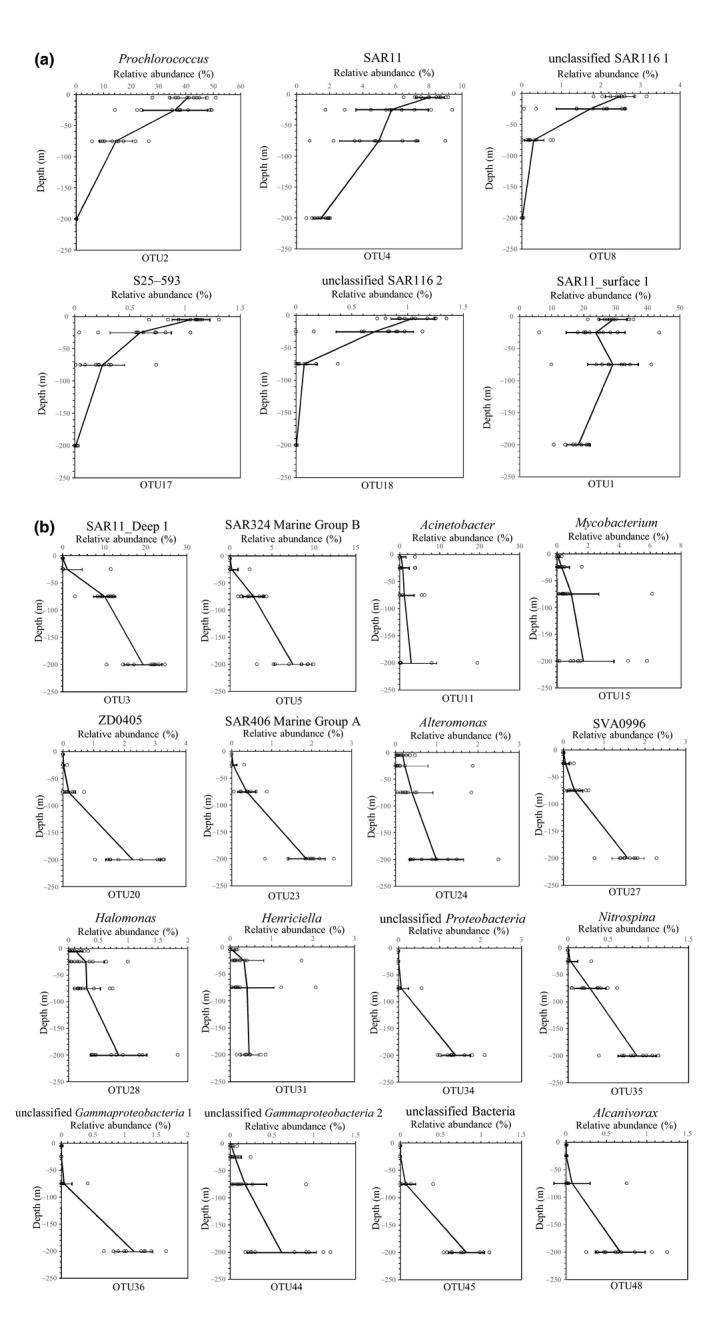
Distributions of the 22 selected dominant operational taxonomic units (OTUs) across depths. Each panel shows the vertical distribution of the relative abundances of each OTU (mean ± one standard deviation) across all 11 sampling stations. The open circles indicate the samples

As demonstrated above, the relative abundances of the major bacterial phyla and classes increased or decreased with depth. Furthermore, when we evaluated the bacterial species, we found that 22 of the 36 dominant OTUs (Data S1), which comprised >1% of the total reads in at least two samples, were vertically stratified. For instance, the relative abundances of six major OTUs, which belong to *Prochlorococcus*, SAR11_surface 1, unclassified SAR11, SAR116, and *Alphaproteobacteria* S25‐593, decreased with depth (Figure 4a). At the same time, the relative abundances of 16 dominant OTUs increased with increasing depth, and these units were affiliated with SAR11_Deep 1, SAR324 Marine Group B, *Acinetobacter*,* Mycobacterium*,* Gammaproteobacteria* ZD0405, SAR406 Marine Group A, *Alteromonas*, marine group *Actinobacteria* (SVA0996), *Halomonas*,* Henriciella*,* Nitrospina*,* Alcanivorax*, unclassified *Proteobacteria*, unclassified *Gammaproteobacteria*, and unclassified bacteria (Figure 4b).

### 3.4 Comparison of the bacterial communities at the eastern and western stations

The hydrographic physical and chemical characteristics revealed the environmental conditions that were distinct between the eastern stations and the western stations, especially at the depth of 75 m. These differences were related to the tilt of the thermocline (Table S1, Figures S1 and S2). Although the bacterial richness and diversity were uniform in each epipelagic layer across the 14°N transect, the community structures were significantly different between the eastern stations (1–4) and the western stations (5–11) at 75 m (Global *R* = .344, *p *= .03).

**Figure 5:**
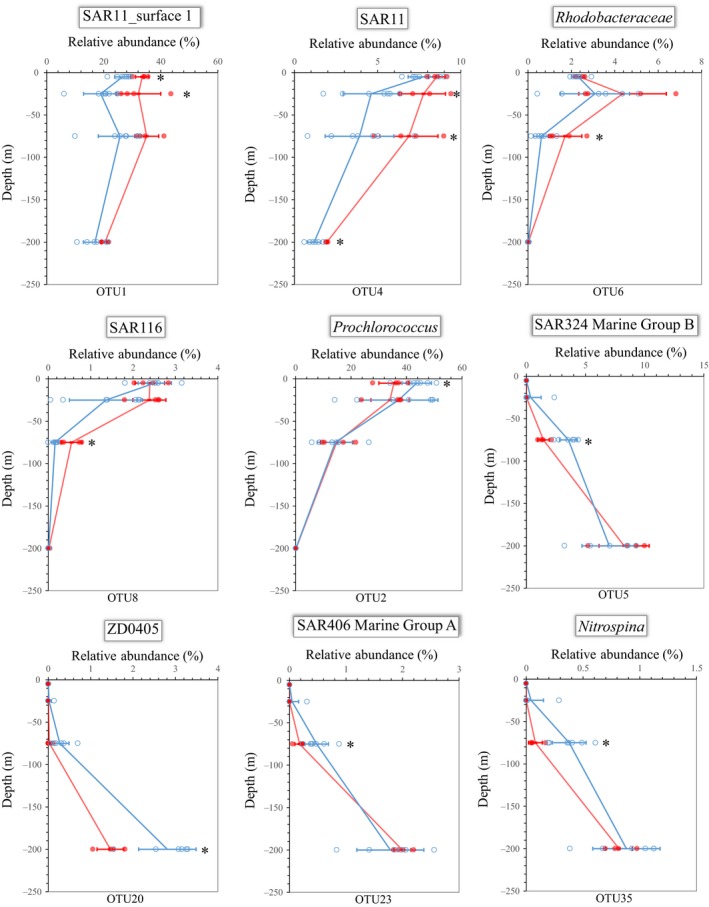
Distributions of the nine selected dominant operational taxonomic units (OTUs), which differed significantly in relative abundances between stations 5–11 and stations 1–4 at different depths. Each panel includes the depth distribution of the OTU relative abundance (mean ± one standard deviation) across the western (blue; open circles) and eastern (red; closed circles) sampling stations. An asterisk indicates that the relative abundances of the OTU were significantly different between the western and the eastern stations

Among the dominant OTUs, the relative abundances of the OTUs affiliated with SAR324 Marine Group B, SAR406 Marine Group A, and *Nitrospina* were much higher in the samples collected from stations 5–11 than in those from stations 1–4 at 75 m (Figure 5). The OTUs belonging to *Prochlorococcus* and ZD0405 (*Oceanospirillales*) were significantly enriched in the communities in the western area at the 5 m and 200 m depths, respectively (Figure 5). In reverse, OTU1 belonging to SAR11_Surface 1 was significantly less abundant at depths of 5 and 25 m at the western stations. OTU4 identified as unclassified SAR11 was significantly less abundant at multiple depth layers, except at 5 m, at stations 5–11 than at stations 1–4 (Figure 5). Additionally, the relative abundances of OTU6 (*Rhodobacteraceae*) and OTU8 (SAR116) were significantly lower in the communities in the western area at 75 m (Figure 5).

### 3.5 Eddy perturbation of the bacterial community

According to the sea‐level anomaly data (Table S2, Figure 1), sampling sites 6–8 were adjacent to a mesoscale cold eddy, and sampling sites 10 and 11 were outside the eddy. Therefore, we compared the bacterial communities between these sites to investigate the effects of the mesoscale eddy. The result of the ANOSIM indicated that no significant differences were observed in the bacterial communities at each depth (*p *> .33), which suggested that the effect of the eddy perturbation on the bacterial communities at these sampling sites was limited.

### 3.6 Environmental drivers of community composition

**Figure 6:**
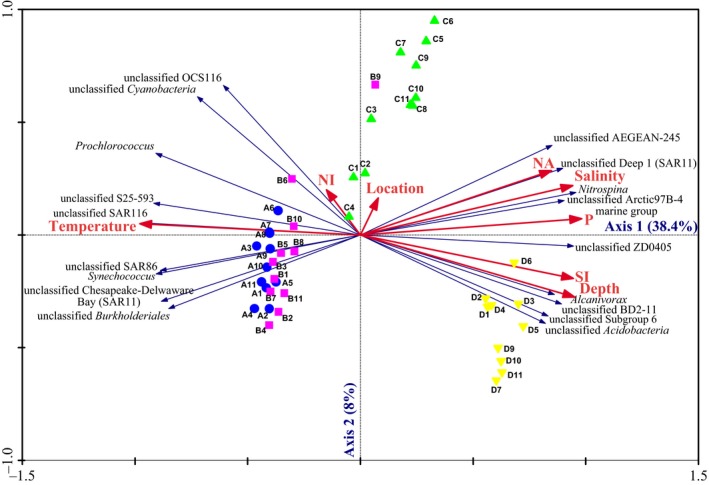
Redundancy analysis ordination of community compositions and environmental variables. The triplot shows the relationship among the spatial and environmental variables, bacterial communities, and the specific bacterial genus components. Only specific bacteria genera with fit values >80% were shown in the triplot. NA, nitrate; NI, nitrite; P, phosphate; SI, silicate. (A) Represents 5 m depth; (B) represents 25 m depth; (C) represents 75 m depth; (D) represents 200 m depth; the numbers 1–11 represent the stations

A redundancy analysis was performed to illuminate the correlation between the variation in the microbial communities and the physicochemical parameters. As shown in Figure 6, along the first axis, the bacterial assemblages in the upper epipelagic layers (5 and 25 m) were clearly distinguished from those in the subsurface layers (75 and 200 m), and the bacterial communities at 75 and 200 m were separated along the second axis (Figure 6). Both the bacterial genera (fit value >80%) and the environmental variables, including location, depth, temperature, salinity, nitrate, nitrite, phosphate, and silicate, were considered in this analysis. The result showed that 56.2% of the total variance in the bacterial compositions could be explained by the environmental parameters included in this study. Thirty‐eight percent of the variance was explained by the first axis with a high species–environment correlation (0.969), and 8% was explained by the second axis with a species–environment correlation value of 0.919 (Table S3, Figure 6). Of all the spatial and physicochemical environmental factors analyzed, phosphate, depth, location, and salinity contributed significantly to the variance of the bacterial community (Table S4). Moreover, the RDA indicates that the grouping based on depth is stronger than that based on geographic location, further pointing to stronger changes in environmental factors along the depth gradient compared to the changes along the longitudinal gradient. A triplot revealed the correlation between the physicochemical factors and the bacterial genera with fit values above 80%. The group unclassified SAR116 was positively correlated with temperature, while the group unclassified ZD0405 (*Oceanospirillales*) was negatively correlated with temperature (Figure 6). *Alcanivorax* was highly positively correlated with depth; in contrast, *Prochlorococcus* was negatively correlated with depth (Figure 6). *Nitrospina* was highly positively correlated with salinity. Unclassified SAR11_Deep 1 was positively correlated with nitrate concentration.

### 3.7 Functional variability

**Figure 7:**
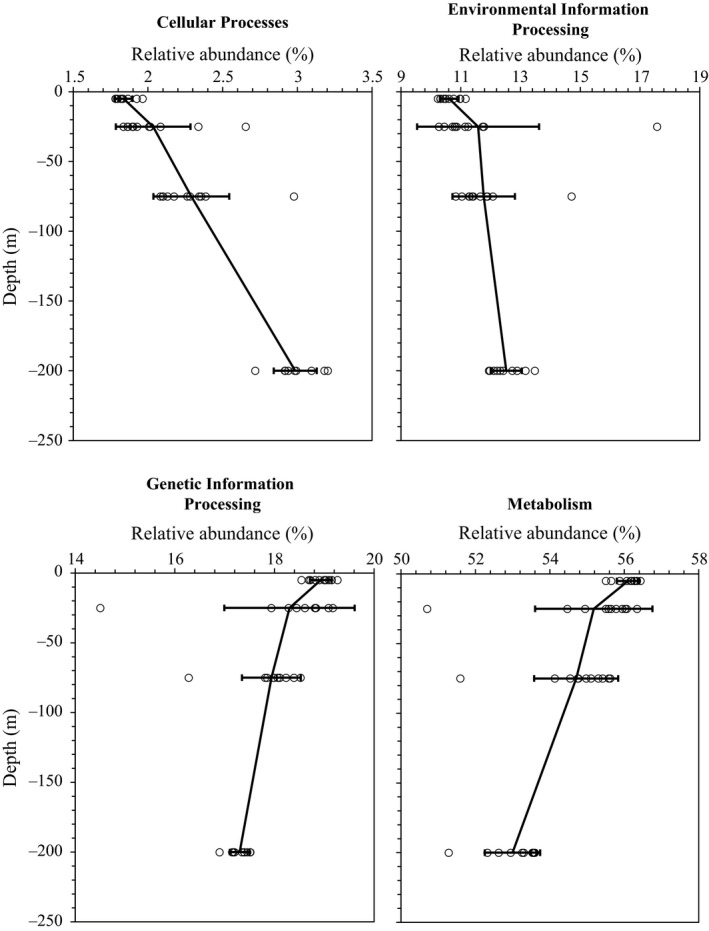
Distributions of the four functional gene categories in bacterioplankton at different depths. Each panel shows the depth distribution of each functional gene category (relative abundance, mean ± one standard deviation) across all 11 sampling stations. The open circles indicate the samples

Through the PICRUSt analysis, metagenomic functional predictions were assigned to KEGG Orthology (KO) tier 3 for all genes with a nearest sequenced taxon index (NSTI) value of 0.056 ± 0.025. Most genes (>50%) were related to metabolism at tier 1 KO categories (Figures 7 and S5). The relative abundances of the functional genes related to metabolism and genetic information processing were higher at the surface than in the deeper layers (Figure 7). Conversely, the genes involved in environmental information processing and cellular processes were enriched with increasing depth (Figure 7). The differences in the relative abundances of these genes at various depths are shown in detail in Table S5. The relative abundances of the genes categorized in human disease and organismal systems were rare with average values of 1.35% and 0.69%, respectively. As the “organismal systems” and “human disease” categories were thought to be poorly relevant to the environmental samples (Staley et al., 2014), they were not further analyzed. On average, 11.17% of the predicted genes could not be annotated (Figure S5).

**Figure 8:**
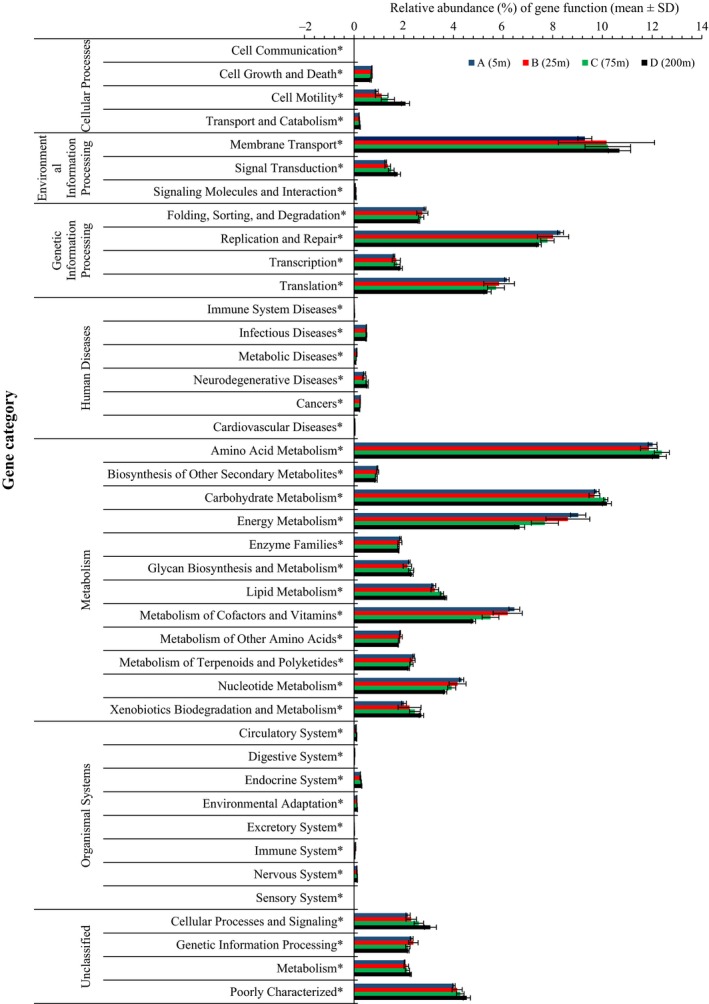
Predicted functions of the epipelagic bacterial communities. The depth distributions of each class of the gene function (relative abundance, mean ± one standard deviation) across all 11 sampling stations are included. An asterisk indicates that the relative abundances of the functional categories are significantly different (Kruskal–Wallis test, *p* < .05) among the four depths

A total of 41 tier 2 KO groups were categorized in the PICRUSt data. The one‐way analysis based on the functional gene compositions also showed vertical stratification (Global *R* = .64, *p *= .001) as well as the taxonomic compositions. Furthermore, all gene families showed statistically significant differences (Kruskal–Wallis test, *p *< .05) among the different depths (Figure 8). A multiple comparison test showed that the differences were mainly between the surface and deeper communities (75 and 200 m) and between the communities at 25 m and the deeper communities (Table S6). We found that the genes related to cell communication, motility, membrane transport, signal transduction, transcription, lipid metabolism and xenobiotics biodegradation and metabolism were enriched with depth (Figure 8). In contrast, the relative abundances of the functional genes involved in nucleotide metabolism, terpenoid and polyketide metabolism, cofactor and vitamin metabolism, energy metabolism, translation, gene replication and repair, and genetic materials folding, sorting, and degradation decreased with depth (Figure 8). Further, the relative abundances of several abundant tier 3 KO groups (exceeding 1% of the total genes in at least two samples; Data S2) varied with increasing depth. Although the functional gene compositions at tier 3 differed at various depths (ANOSIM analysis Global *R* = .652, *p *= .001), they were found to be similar across the 11 sampling profiles at each depth layer, even when the functional compositions at the eastern stations were compared with those at the western stations (*p* > .1) and at stations 6–8 and 10–11 (*p *> .667).

## 4 DISCUSSION

The ocean is the largest ecosystem on the Earth and is inhabited by ample plankton taxa; the majority of these taxa are unexplored, and little is known about them (Bork et al., 2015; Rappé & Giovannoni, 2003). A comprehensive understanding of the bacterioplankton distributions and their responses to dynamic environments is critical for understanding their ecological roles. This study evaluated the epipelagic bacterial communities at the basin scale in the context of the spatial and physicochemical environment in the SCS. Moreover, we reached a preliminary understanding of the distribution of the functional genes in the epipelagic zone in the SCS by predicting the functional compositions.

### 4.1 Depth stratification of bacteria in the SCS

This study revealed that the bacterial species richness and diversity increased with depth in the epipelagic zone in the SCS. This result is consistent with the results reported by Sunagawa et al. (2015) at the global scale. Depth partitioning in the distribution of prokaryotic communities has also been reported previously at a few stations in the SCS (Cai & Jiao, 2008; Hu, Jiao, & Zhang, 2011; Tseng et al., 2015; Zhang, Zhao et al., 2011). In this study, we revealed that the bacterial communities were vertically stratified across multiple depths along the 14°N transect in the SCS. To the best of our knowledge, this is the first study to show bacterial stratification at the basin scale in the SCS.

Several major OTUs with high relative abundances represented signatures specific to water depth in the bacterial communities. For example, the SAR11 clade is known as dominant heterotrophs in oligotrophic marine ecosystems (Morris, Frazar, & Carlson, 2012), and it has been divided into surface and deep subclades (Vergin et al., 2013). Our results clearly support the previous reports of the vertical distribution of SAR11 (Carlson et al., 2009; Field et al., 1997). The relative abundances of the OTUs affiliated with ZD405 (*Oceanospirillales*), SAR406, and SAR324 Marine Group B were found to increase with depth in the Atlantic and Pacific oceans in previous studies (AgoguÉ, Lamy, Neal, Sogin, & Herndl, 2011; Gordon & Giovannoni, 1996; Wright, Vergin, Boyd, & Giovannoni, 1997), and these OTUs also exhibited markedly increasing relative abundances with increasing depth in the SCS. Therefore, we suggest that the OTUs affiliated with the ZD405 (*Oceanospirillales*), SAR406, and SAR324 clades, which were proposed as reliable tracers of water masses originating from the mesopelagic realm in the Sargasso Sea (Nelson et al., 2014), might also be applicable to tracing water masses in the SCS.

Consistent with previous results (DeLong et al., 2006), the OTUs affiliated with *Prochlorococcus*, SAR116, and an unclassified SAR11 clade were abundant in the surface waters, and their abundances declined with depth. Different phylogenetic groups within the SAR11 clade are found in different regions of the water column (Field et al., 1997; Morris et al., 2002). Thus, OTU4, which belongs to the unclassified SAR11 clade and showed decreasing abundance with depth, should be the surface ecotype.

At the SouthEast Asia Time‐series Study station, the prokaryotic metagenomes showed that membrane transport genes were enriched at a depth of 100 m, and signal transduction genes were more abundant at 1,000 m than at 10 and 3,000 m (Tseng et al., 2015). Similarly, these two categories of genes were proven to be enriched in the deeper epipelagic layers in the SCS in this study. Moreover, the relative abundance of the functional genes related to cell motility, transcription, and sensory system also increased with depth. This result is probably because the abundance of bacteria with copiotrophic lifestyles increased with depth, and they respond to environmental stimuli and acquire nutrients through these functional genes expression (Lauro et al., 2009).

### 4.2 Spatial distribution of bacterial community along a longitudinal transect

Recent reports demonstrated the longitudinal distribution of planktonic marine bacteria across the Mediterranean Sea (Mapelli et al., 2013) and from the east Atlantic to the Indian Ocean (Haggerty & Dinsdale, 2017). In this study, the distribution of bacterial communities was first investigated across the entire basin along the latitude 14°N in the SCS. In mid‐autumn (October), the northeasterly monsoon prevails south of 12°N, and the sea surface temperature (SST) decreases in the north of the SCS. In particular, the SST east of Vietnam, where the western boundary current driven by the monsoon allows cold water to upwell to the surface, is distinctly lower than in other parts of the SCS (Liu et al., 2004). The sea surface height is higher in the eastern SCS and lower east of Vietnam (Li, Xu, Jing, Wu, & Guo, 2003). Similar to the climatological mean, high sea levels were observed in the eastern SCS, while low sea levels were observed in the western SCS during the cruise (Figure 1). The vertical profile of the temperature along 14°N demonstrated that the thermocline was deeper in the eastern SCS than in the western SCS (Figure S1). Consistent with the distinct physicochemical environments in the eastern and western SCS across the basin, differences in the bacterial assemblages were confirmed between the east and west areas, especially at the depths of 75 m across the longitudinal transect. Moreover, the distributions of several dominant OTUs also revealed the localized responses. For instance, the OTUs affiliated with SAR116 and SAR11, which are oligotrophs that inhabit the surface water and have metabolic adaptations to low‐nutrient, high‐light environments (Giovannoni & Stingl, 2005; Oh et al., 2010; Vergin et al., 2013), were significantly more abundant in the east than in the west at 75 m. This distribution may result from the deeper thermocline in the east than in the west. Additionally, no significant differences were observed in the functional gene compositions between the east and west areas in the 75‐m layer. This observation supports the idea that the biogeography patterns in bacterial communities differ from the taxa or function perspective (Haggerty & Dinsdale, 2017).

### 4.3 Effects of eddy on the bacterial community

As mesoscale eddies were found adjacent to the sampling profile, we evaluated the bacterial communities within and outside the cyclonic eddy, and significant differences were not observed in taxonomic compositions or functional gene compositions. In previous reports, the eddy center and eddy edge were defined as the locations <20 and >25 km from the point of minimum velocity, respectively, based on the distance or clustered according to the potential density and dissolved inorganic nitrogen concentrations (Ewart, Meyers, Wallner, McGillicuddy, & Carlson, 2008; Nelson et al., 2014). Nelson et al. (2014) further observed that the bacterioplankton communities at the eddy edge were similar to those at the outside comparative station, which was not affected by the eddy. Accordingly, the results obtained in this study suggest that the effect of the mesoscale eddy on the bacterial community was limited due to the large distances (>300 km) between the sampling stations and the center of the eddy.

### 4.4 Environmental drivers of bacterial community distribution

In addition to the spatial factor, the environmental parameters, phosphate, and salinity appeared to contribute to the variation in the bacterial communities in the SCS. This finding provided new evidence to support the hypothesis about biogeographic patterns of bacterial assemblages that the community structure reflects environmental dissimilarity (Haggerty & Dinsdale, 2017). In agreement with the results obtained in this study, previous research demonstrated that the availability of phosphorus plays important roles in planktonic growth and activity, as was demanded by heterotrophic bacteria in oligotrophic marine environments (Fuller et al., 2005; Moutin et al., 2002; Van Mooy, Rocap, Fredricks, Evans, & Devol, 2006). At the same time, salinity may act as a barrier separating bacterial populations (Bouvier & del Giorgio, 2002; Pommier et al., 2007) and therefore has a substantial effect on the bacterial community structure.

### 4.5 Functional genes from the PICRUSt prediction

Although only marker gene surveys were currently available and the predictions were somewhat limited by the sequencing depths, PICRUSt provided useful insights and generally accurate functional findings related to the functional capabilities of the community even at a shallow 16S rRNA gene sequencing depth (Langille et al., 2013). The NSTI was used to quantify the availability of nearby genome representatives for each microbiome sample, with lower values indicating a closer mean relationship between the microbes in a given sample and the microbes with sequenced genome representatives (Langille et al., 2013; Staley et al., 2014). The NSTI values for the samples analyzed in this study indicated the suitability of the predictions from PICRUSt. We have discussed the similar vertically stratified distributions of the gene functions and the bacterial taxonomic community, and the inconsistent distribution patterns of these in the eastern and western stations across the longitudinal transect in the above content. These results emphasize that further investigation and verification of the functional characteristics of bacterial components would be helpful for understanding their biogeographic distribution and ecological roles in nature.

## 5 CONCLUSION

This study demonstrated the basin‐scale variability in the epipelagic bacterioplankton community across the 14°N transect in the SCS. Our results revealed the vertical stratification of both bacterial taxa and their potential functions in the epipelagic zone. The effects of the spatial and physicochemical environmental conditions in the eastern and western SCS on the epipelagic community composition were confirmed, and a significant influence was observed at a depth of 75 m. To the best of our knowledge, this is the first attempt to elucidate the bacterial community in the SCS at the basin scale and simultaneously discuss the impacts of physical forces, including differences between the east–west environments driven by the monsoon at the basin scale and eddies at the mesoscale. Given the complexity of the environment driven by irregular and frequent multiscale physical forces in the SCS, the investigation of the variation in the microbial community associated with ocean dynamics in the SCS through multidisciplinary cooperation will help us understand the dynamic microbial assemblages and their ecological significance.

## CONFLICT OF INTEREST

None declared.

## AUTHOR CONTRIBUTIONS

Si Zhang and Jie Li designed this study. Yi Zhang, Jie Li, and Xuhua Cheng analyzed the data. Yi Zhang, Jie Li, and Xuhua Cheng wrote the manuscript. Zhimao Mai collected the seawater samples on board. Yinfeng Luo provided technical sequencing assistance. All authors commented on the manuscript and made suggestions.

### DATA ACCESSIBILITY

The pyrosequencing data have been deposited and are publicly available in the NCBI Sequence Read Archive (SAR). The accession number is SRR3744867. Data S1, the abundant OTU table; Data S2, the abundant tier 3 KO group table. The modules used for the functional prediction in this work are publicly available at http://huttenhower.sph.harvard.edu/galaxy/root/index
