# Supplementary figures and images for: Community differentiation of bacterioplankton in the epipelagic layer in the South China Sea
Source: Ecol Evol. 2018 Apr 19;8(10):4932–48. doi: 10.1002/ece3.4064 (PMC5980402; doi:10.1002/ece3.4064)

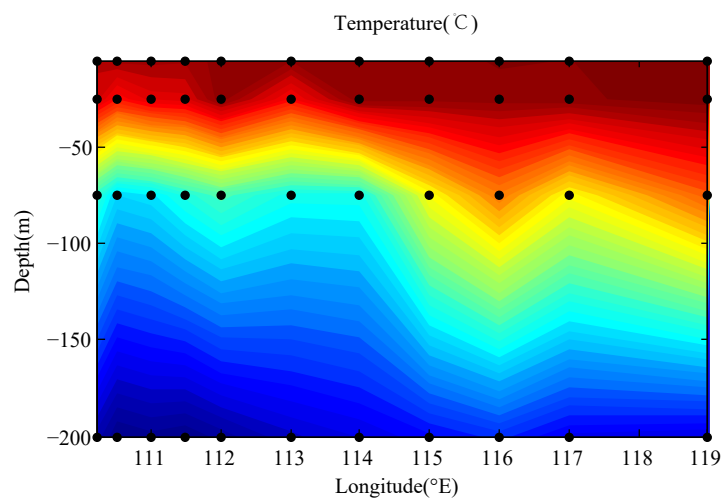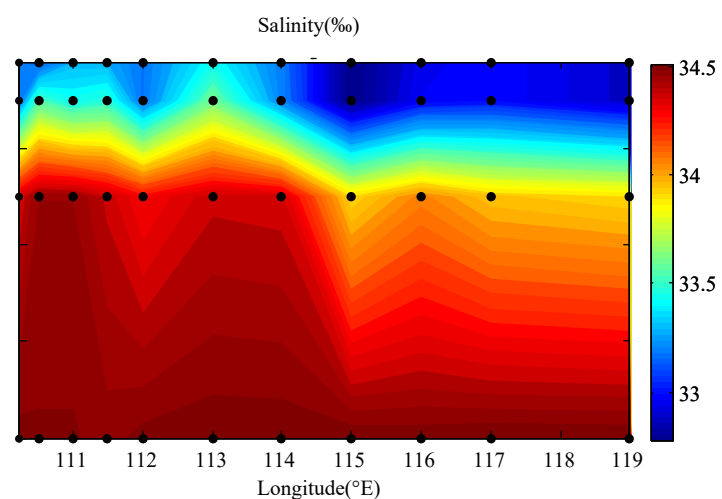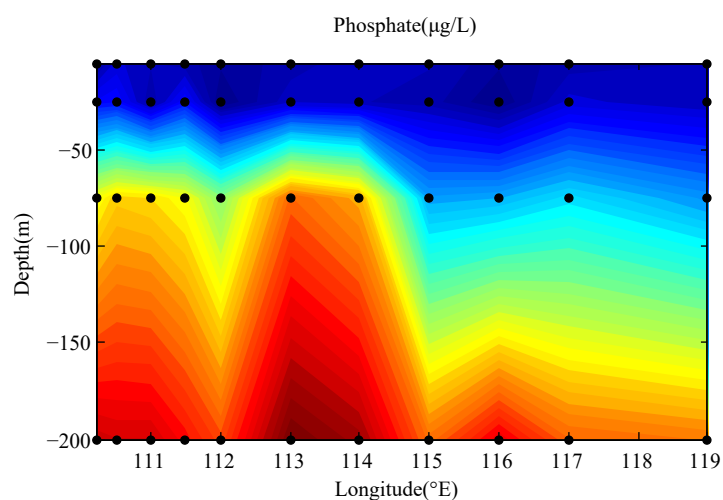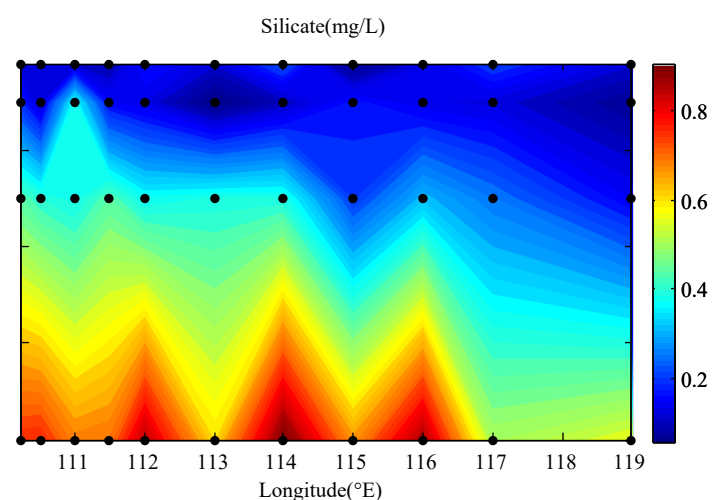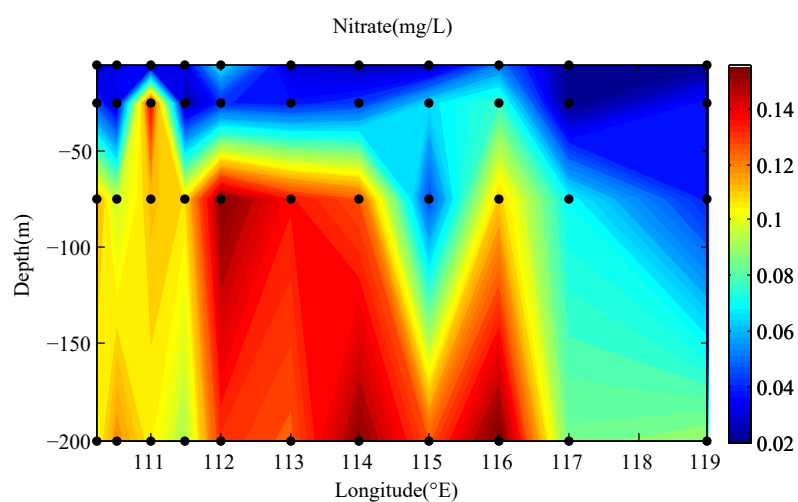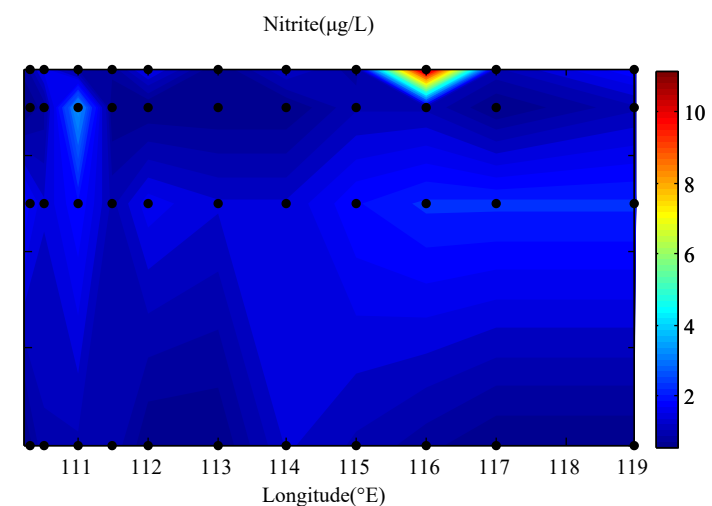

Supplement: Supplementary file 1 [file ECE3-8-4932-s001.pdf]

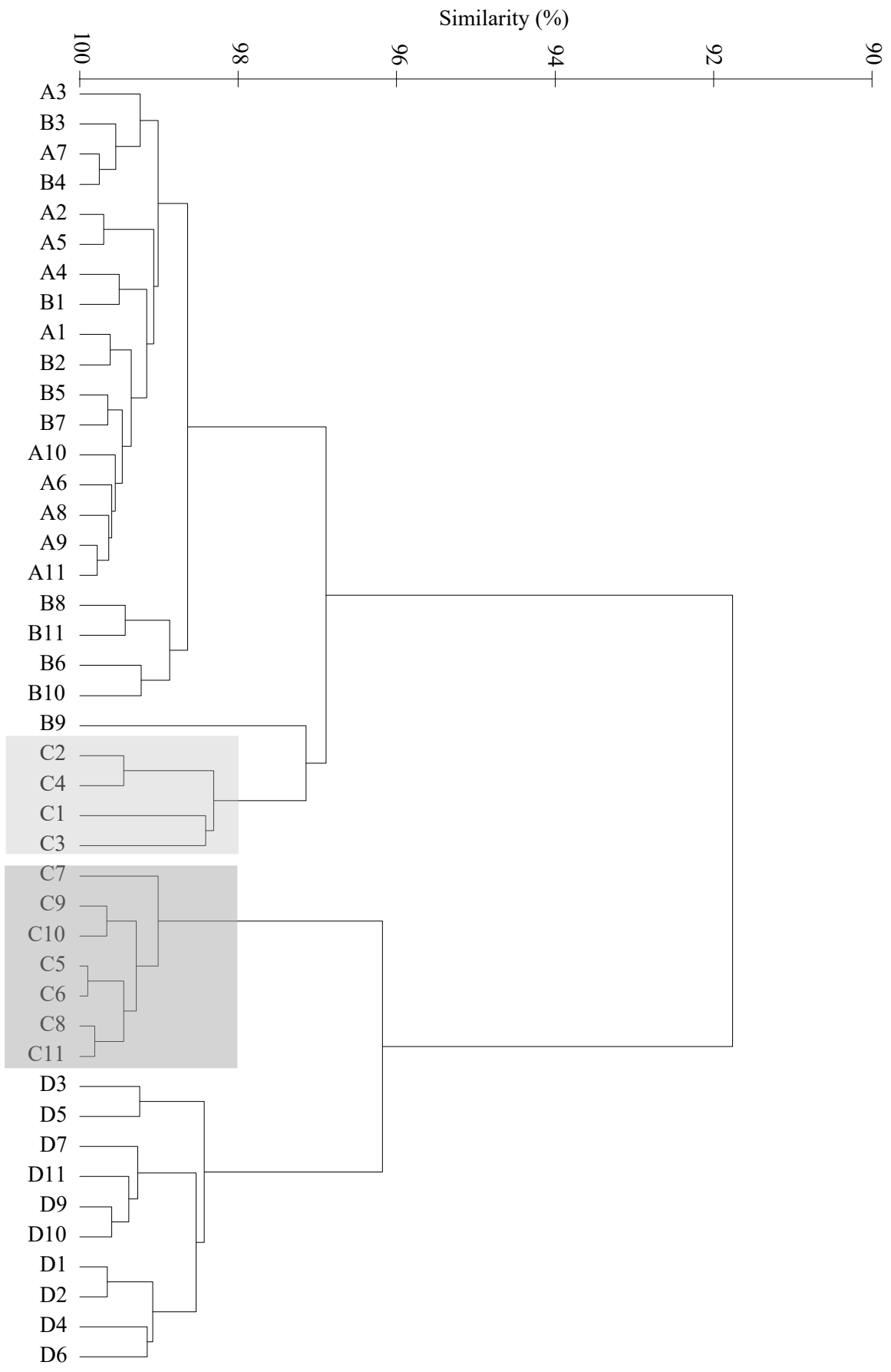

Supplement: Supplementary file 2 [file ECE3-8-4932-s002.pdf]

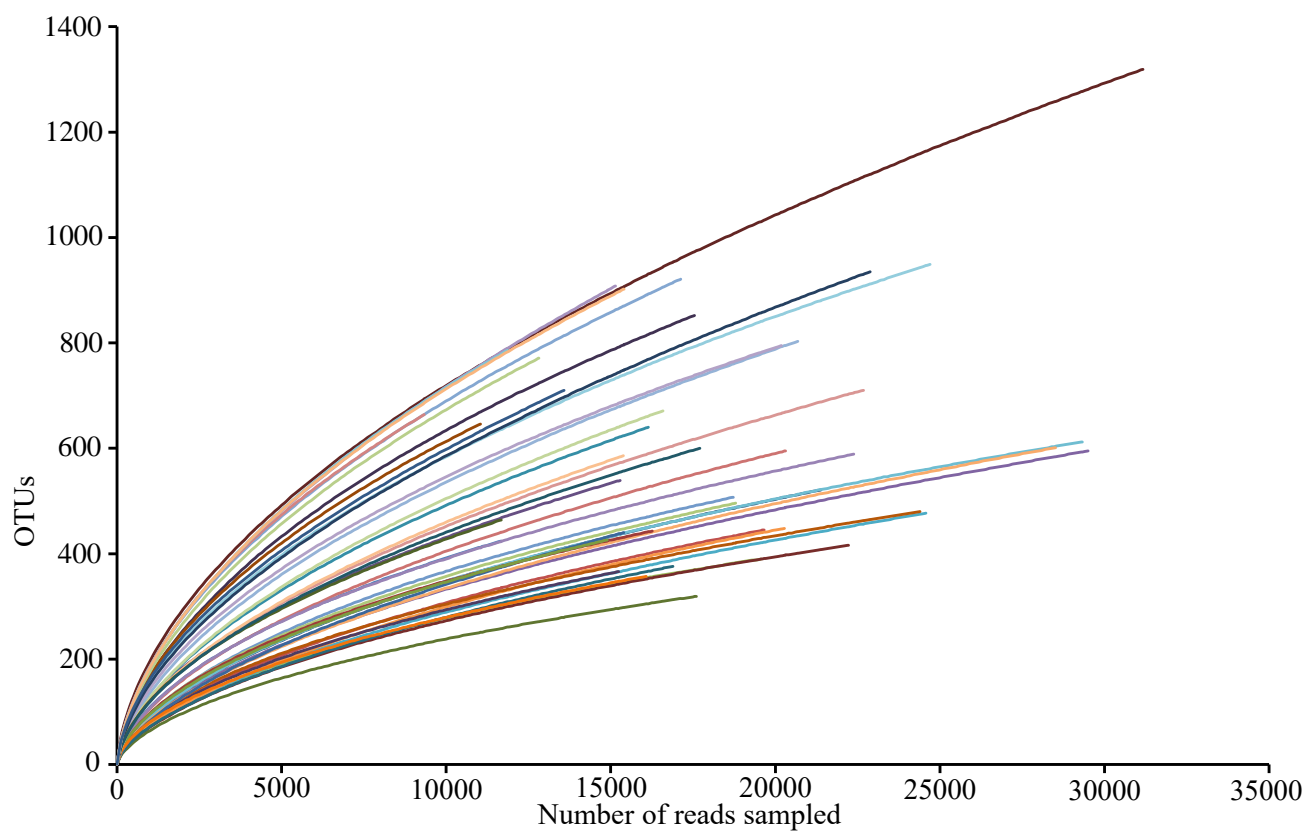

|    |     |     |    |    |    |    |    |    |    |    |
|----|-----|-----|----|----|----|----|----|----|----|----|
| A1 | A10 | A11 | A2 | A3 | A4 | A5 | A6 | A7 | A8 | A9 |
| B1 | B10 | B11 | B2 | B3 | B4 | B5 | B6 | B7 | B8 | B9 |
| C1 | C10 | C11 | C2 | C3 | C4 | C5 | C6 | C7 | C8 | C9 |
| D1 | D10 | D11 | D2 | D3 | D4 | D5 | D6 | D7 | D9 |    |

Supplement: Supplementary file 3 [file ECE3-8-4932-s003.pdf]

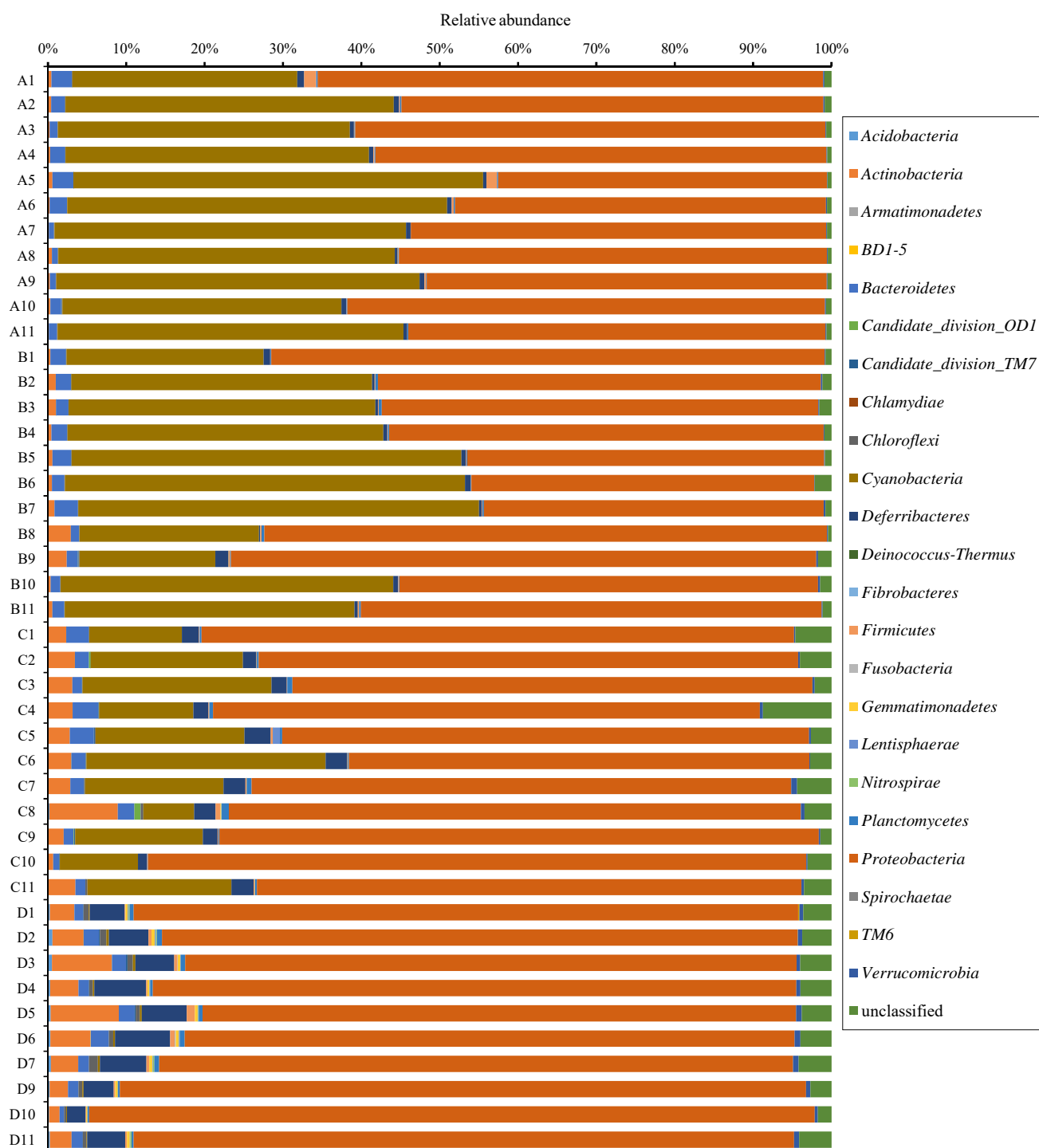

Supplement: Supplementary file 4 [file ECE3-8-4932-s004.pdf]

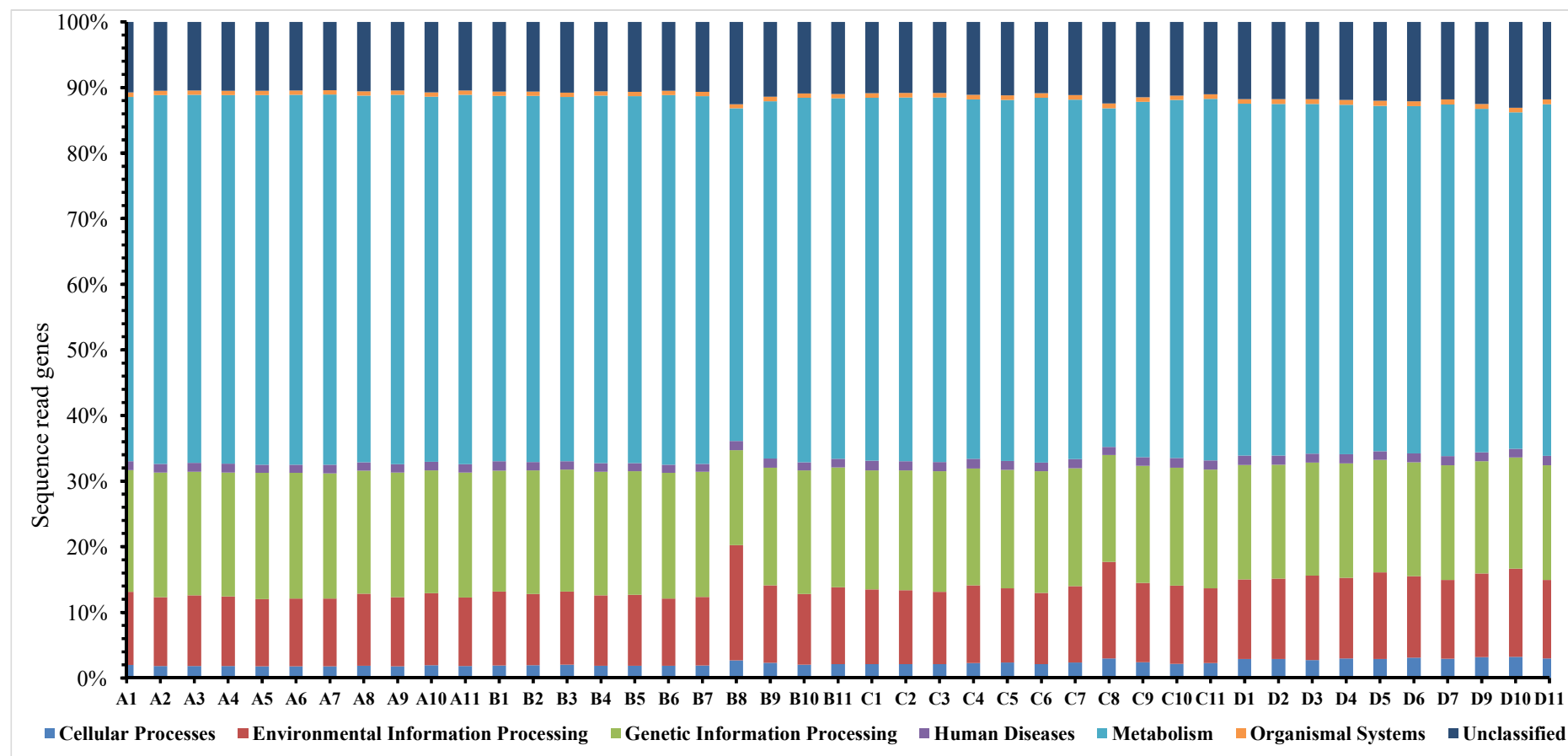

Supplement: Supplementary file 5 [file ECE3-8-4932-s005.pdf]
