# Supplementary material for: Community differentiation of bacterioplankton in the epipelagic layer in the South China Sea
Source: Ecol Evol. 2018 Apr 19;8(10):4932–48. doi: 10.1002/ece3.4064 (PMC5980402; doi:10.1002/ece3.4064)
Supplement: Supplementary file 6 [file ECE3-8-4932-s006.doc]

**Supplementary tables and figures**

**Table S1.** Physicochemical parameters of the seawater samples collected from the South China Sea. The location was identified by calculating the longitude-based relative distance from sampling station no.1.

| Sample | Longitude (°) | Latitude (°) | Salinity (‰) | Temperature (℃) | Phosphate (μg/L) | Nitrite (μg/L) | Nitrate (mg/L) | Silicate (mg/L) | Depth （m） | Location | Station |
| --- | --- | --- | --- | --- | --- | --- | --- | --- | --- | --- | --- |
| A1 | 119.00 | 14.00 | 32.90 | 28.93 | 3.52 | 2.04 | 0.022 | 0.129 | 5 | 0 | 1 |
| A2 | 117.00 | 14.00 | 33.02 | 28.62 | 3.03 | 1.17 | 0.022 | 0.213 | 5 | 2 | 2 |
| A3 | 116.00 | 14.00 | 32.97 | 28.67 | 2.05 | 11.36 | 0.053 | 0.141 | 5 | 3 | 3 |
| A4 | 115.00 | 14.00 | 32.78 | 28.46 | 3.52 | 0.87 | 0.026 | 0.063 | 5 | 4 | 4 |
| A5 | 114.00 | 14.00 | 33.21 | 28.54 | 4.01 | 1.46 | 0.027 | 0.231 | 5 | 5 | 5 |
| A6 | 113.00 | 14.00 | 33.45 | 28.41 | 3.03 | 0.58 | 0.031 | 0.129 | 5 | 6 | 6 |
| A7 | 112.00 | 14.00 | 33.19 | 28.49 | 2.05 | 1.75 | 0.069 | 0.183 | 5 | 7 | 7 |
| A8 | 111.50 | 14.00 | 33.30 | 28.08 | 3.03 | 0.87 | 0.035 | 0.099 | 5 | 7.5 | 8 |
| A9 | 111.00 | 14.00 | 33.31 | 28.13 | 3.52 | 0.87 | 0.027 | 0.123 | 5 | 8 | 9 |
| A10 | 110.50 | 14.00 | 33.13 | 28.12 | 4.50 | 1.75 | 0.038 | 0.135 | 5 | 8.5 | 10 |
| A11 | 110.24 | 14.00 | 33.20 | 27.84 | 2.54 | 0.87 | 0.025 | 0.123 | 5 | 8.76 | 11 |
| B1 | 119.00 | 14.00 | 32.90 | 28.84 | 3.52 | 1.17 | 0.041 | 0.075 | 25 | 0 | 1 |
| B2 | 117.00 | 14.00 | 33.02 | 28.55 | 4.50 | 0.58 | 0.020 | 0.135 | 25 | 2 | 2 |
| B3 | 116.00 | 14.00 | 32.99 | 28.50 | 1.08 | 1.17 | 0.079 | 0.147 | 25 | 3 | 3 |
| B4 | 115.00 | 14.00 | 32.78 | 28.46 | 3.03 | 1.17 | 0.067 | 0.183 | 25 | 4 | 4 |
| B5 | 114.00 | 14.00 | 33.22 | 28.55 | 3.52 | 0.58 | 0.044 | 0.111 | 25 | 5 | 5 |
| B6 | 113.00 | 14.00 | 33.63 | 26.73 | 4.01 | 0.58 | 0.040 | 0.057 | 25 | 6 | 6 |
| B7 | 112.00 | 14.00 | 33.19 | 28.49 | 1.08 | 0.58 | 0.042 | 0.141 | 25 | 7 | 7 |
| B8 | 111.50 | 14.00 | 33.50 | 27.34 | 6.46 | 0.58 | 0.025 | 0.165 | 25 | 7.5 | 8 |
| B9 | 111.00 | 14.00 | 33.45 | 27.25 | 2.85 | 3.50 | 0.139 | 0.387 | 25 | 8 | 9 |
| B10 | 110.50 | 14.00 | 33.57 | 26.57 | 6.46 | 0.87 | 0.030 | 0.123 | 25 | 8.5 | 10 |
| B11 | 110.24 | 14.00 | 33.21 | 27.50 | 5.97 | 0.58 | 0.046 | 0.195 | 25 | 8.76 | 11 |
| C1 | 119.00 | 14.00 | 33.93 | 25.52 | 11.84 | 2.33 | 0.040 | 0.159 | 75 | 0 | 1 |
| C2 | 117.00 | 14.00 | 33.97 | 23.03 | 14.77 | 2.33 | 0.071 | 0.261 | 75 | 2 | 2 |
| C3 | 116.00 | 14.00 | 34.08 | 24.99 | 12.82 | 2.33 | 0.112 | 0.321 | 75 | 3 | 3 |
| C4 | 115.00 | 14.00 | 33.93 | 23.00 | 12.33 | 2.04 | 0.046 | 0.207 | 75 | 4 | 4 |
| C5 | 114.00 | 14.00 | 34.39 | 19.50 | 28.96 | 1.46 | 0.128 | 0.399 | 75 | 5 | 5 |
| C6 | 113.00 | 14.00 | 34.40 | 19.47 | 31.89 | 1.46 | 0.137 | 0.399 | 75 | 6 | 6 |
| C7 | 112.00 | 14.00 | 34.30 | 20.39 | 21.13 | 1.75 | 0.156 | 0.381 | 75 | 7 | 7 |
| C8 | 111.50 | 14.00 | 34.40 | 19.75 | 25.53 | 1.17 | 0.109 | 0.447 | 75 | 7.5 | 8 |
| C9 | 111.00 | 14.00 | 34.48 | 18.95 | 27.00 | 2.04 | 0.113 | 0.387 | 75 | 8 | 9 |
| C10 | 110.50 | 14.00 | 34.48 | 18.73 | 27.49 | 1.46 | 0.097 | 0.411 | 75 | 8.5 | 10 |
| C11 | 110.24 | 14.00 | 34.38 | 19.93 | 25.53 | 2.04 | 0.116 | 0.447 | 75 | 8.76 | 11 |
| D1 | 119.00 | 14.00 | 34.55 | 15.13 | 30.91 | 0.58 | 0.091 | 0.567 | 200 | 0 | 1 |
| D2 | 117.00 | 14.00 | 34.55 | 15.35 | 31.89 | 0.58 | 0.086 | 0.513 | 200 | 2 | 2 |
| D3 | 116.00 | 14.00 | 34.54 | 16.14 | 36.29 | 0.87 | 0.158 | 0.878 | 200 | 3 | 3 |
| D4 | 115.00 | 14.00 | 34.55 | 15.63 | 31.40 | 1.17 | 0.128 | 0.674 | 200 | 4 | 4 |
| D5 | 114.00 | 14.00 | 34.54 | 15.13 | 39.23 | 1.46 | 0.154 | 0.920 | 200 | 5 | 5 |
| D6 | 113.00 | 14.00 | 34.54 | 14.73 | 41.19 | 0.58 | 0.127 | 0.615 | 200 | 6 | 6 |
| D7 | 112.00 | 14.00 | 34.53 | 14.04 | 32.87 | 0.58 | 0.133 | 0.860 | 200 | 7 | 7 |
| D9 | 111.00 | 14.00 | 34.52 | 13.81 | 37.27 | 1.17 | 0.107 | 0.692 | 200 | 8 | 9 |
| D10 | 110.50 | 14.00 | 34.52 | 13.62 | 37.27 | 1.17 | 0.120 | 0.770 | 200 | 8.5 | 10 |
| D11 | 110.24 | 14.00 | 34.53 | 14.36 | 37.76 | 0.58 | 0.104 | 0.764 | 200 | 8.76 | 11 |

**Table S2.** Values of the sea level anomaly.

| Station | Sampling date | Longitude | Latitude | sea level anomaly (cm) |
| --- | --- | --- | --- | --- |
| 1 | Oct. 22, 2012 | 119.00° | 14.00° | 11.3 |
| 2 | Oct. 21, 2012 | 117.00° | 14.00° | 9.5275 |
| 3 | Oct. 21, 2012 | 116.00° | 14.00° | 15.0625 |
| 4 | Oct. 21, 2012 | 115.00° | 14.00° | 11.4375 |
| 5 | Oct. 21, 2012 | 114.00° | 14.00° | -1.8475 |
| 6 | Oct. 20, 2012 | 113.00° | 14.00° | -5.035 |
| 7 | Oct. 20, 2012 | 112.00° | 14.00° | -8.8225 |
| 8 | Oct. 20, 2012 | 111.50° | 14.00° | -6.5175 |
| 9 | Oct. 19, 2012 | 111.00° | 14.00° | 1.0975 |
| 10 | Oct. 19, 2012 | 110.50° | 14.00° | 4.43 |
| 11 | Oct. 19, 2012 | 110.24° | 14.00° | 7.17 |

**Table S3.** Summary of the redundancy analysis with automatic forward selection of the Monta Carlo permutation tests (permutations = 499).

| Axes | 1 | 2 | 3 | 4 | Total variance |
| --- | --- | --- | --- | --- | --- |
| Eigenvalues | 0.384 | 0.080 | 0.042 | 0.020 | 1.000 |
| Species-environment correlations | 0.969 | 0.919 | 0.844 | 0.740 |  |
| Cumulative percentage variance of species data | 38.4 | 46.4 | 50.6 | 52.6 |  |
| Cumulative percentage variance of species-environment relation | 68.4 | 82.6 | 90.1 | 93.6 |  |
| Sum of all eigenvalues |  |  |  |  | 1.000 |
| Sum of all canonical eigenvalues |  |  |  |  | 0.562 |

**Table S4.** Summary of the marginal and conditional effects of the environmental variables with automatic forward selection of the Monta Carlo permutation tests (permutations = 499).

| Marginal Effects | | | Conditional Effects | | | | |
| --- | --- | --- | --- | --- | --- | --- | --- |
| Variable | Var.N | Lambda1 | Variable | Var.N | LambdaA | P | F |
| Phosphate | 3 | 0.37 | Phosphate | 3 | 0.37 | 0.002 | 24.24 |
| Temperature | 2 | 0.37 | Depth | 7 | 0.07 | 0.002 | 4.66 |
| Depth | 7 | 0.35 | Location | 8 | 0.03 | 0.004 | 2.70 |
| Silicate | 6 | 0.35 | Salinity | 1 | 0.04 | 0.008 | 2.60 |
| Salinity | 1 | 0.35 | Nitrate | 5 | 0.01 | 0.264 | 1.15 |
| Nitrate | 5 | 0.28 | Silicate | 6 | 0.02 | 0.168 | 1.36 |
| Location | 8 | 0.04 | Nitrite | 4 | 0.01 | 0.380 | 0.95 |
| Nitrite | 4 | 0.02 | Temperature | 2 | 0.01 | 0.660 | 0.79 |

**Table S5**. Results of the Kruskalmc's multiple comparisons test after Kruskal-Wallis for functional traits at tier 1 KO categories. An asterisk indicates that the relative abundances of the functional categories are significantly different (*P* < 0.05) among the four depths. “True” represents that differences between pairwise groups are statistically different, “False” represents that differences between pairwise groups are not statistically different, at 0.05 significance level.

| Category | Difference | | | | | |
| --- | --- | --- | --- | --- | --- | --- |
|  | 5-25 m | 5-75m | 5-200m | 25-75m | 25-200m | 75-200m |
| Cellular processes* | False | True | True | False | True | False |
| Environmental information processing* | False | True | True | False | True | False |
| Genetic information processing* | False | True | True | False | True | False |
| Metabolism* | False | True | True | False | True | False |

**Table S6.** Results of the Kruskalmc's multiple comparisons test after Kruskal-Wallis for functional traits at tier 2 KO categories. An asterisk indicates that the relative abundances of the functional categories are significantly different (*P* < 0.05) among the four depths. “True” represents that differences between pairwise groups are tatistically different, “False” represents that differences between pairwise groups are not statistically different, at 0.05 significance level.

| Category | Difference | | | | | |
| --- | --- | --- | --- | --- | --- | --- |
|  | 5-25 m | 5-75m | 5-200m | 25-75m | 25-200m | 75-200m |
| Cell Communication* | False | True | True | False | True | False |
| Cell Growth and Death* | False | False | True | False | True | True |
| Cell Motility* | False | True | True | False | True | False |
| Transport and Catabolism* | False | True | True | False | True | False |
| Membrane Transport* | False | True | True | False | True | False |
| Signal Transduction* | False | True | True | False | True | False |
| Signaling Molecules and Interaction* | False | False | True | False | False | True |
| Folding, Sorting and Degradation* | False | True | True | False | True | False |
| Replication and Repair* | False | True | True | False | True | False |
| Transcription* | False | True | True | False | True | False |
| Translation* | False | True | True | False | True | False |
| Immune System Diseases* | False | True | True | False | True | False |
| Infectious Diseases* | False | False | False | False | False | True |
| Metabolic Diseases* | False | True | True | True | True | False |
| Neurodegenerative Diseases* | False | True | True | True | True | False |
| Cancers* | False | False | True | False | True | False |
| Cardiovascular Diseases* | False | True | True | True | False | False |
| Amino Acid Metabolism* | False | True | False | True | True | False |
| Biosynthesis of Other Secondary Metabolites* | False | False | True | True | True | False |
| Carbohydrate Metabolism* | False | True | True | True | True | False |
| Energy Metabolism* | False | True | True | False | True | False |
| Enzyme Families* | False | True | True | True | False | False |
| Glycan Biosynthesis and Metabolism* | False | False | True | True | True | False |
| Lipid Metabolism* | False | True | True | True | True | False |
| Metabolism of Cofactors and Vitamins* | False | True | True | False | True | False |
| Metabolism of Other Amino Acids* | False | False | True | True | True | False |
| Metabolism of Terpenoids and Polyketides* | False | True | True | False | True | False |
| Nucleotide Metabolism* | False | True | True | False | True | False |
| Xenobiotics Biodegradation and Metabolism* | False | True | True | False | True | False |
| Circulatory System* | False | True | True | True | True | False |
| Digestive System* | False | True | True | True | True | False |
| Endocrine System* | False | True | True | True | True | False |
| Environmental Adaptation* | False | True | True | True | True | False |
| Excretory System* | False | True | True | False | True | False |
| Immune System* | False | True | True | True | True | False |
| Nervous System* | False | True | True | True | True | False |
| Sensory System * | False | False | False | False | False | False |
| Cellular Processes and Signaling* | False | True | True | False | True | False |
| Genetic Information Processing* | False | True | True | True | True | False |
| Metabolism* | False | True | True | False | True | False |
| Poorly Characterized* | False | True | True | False | True | False |

**Fig. S1** Depth distributions of the physical and geochemical parameters across all 11 sampling profiles along the 14°N transect. The points represent the sampling stations.

**Fig. S2** Hierarchical cluster dendrogram for all seawater samples generated from the Bray-Curtis similarity of the environmental factors using cluster analysis (CLUSTER in primer 5 software). A represents 5 m depth; B represents 25 m depth; C represents 75 m depth; D represents 200 m depth; the numbers 1–11 represent the stations.

**Fig. S3** Rarefaction curves for the bacterial communities. OTUs were defined at 97% sequence similarity. A represents 5 m depth; B represents 25 m depth; C represents 75 m depth; D represents 200 m depth; the numbers 1–11 represent the stations.

**Fig. S4** Bacterial composition profiles in the 43 seawater samples. Taxonomic classification of the bacterial reads into phylum using the classify.seqs command with the SILVA reference files and a bootstrap confidence level of 80% in Mothur program. A represents 5 m depth; B represents 25 m depth; C represents 75 m depth; D represents 200 m depth; the numbers 1–11 represent the stations.

**Fig. S5** Percentages of the tier 1 KO categories from the PICRUSt functional predictions. A represents 5 m depth; B represents 25 m depth; C represents 75 m depth; D represents 200 m depth; the numbers 1–11 represent the stations.
